# Cancer Acidity and Hypertonicity Contribute to Dysfunction of Tumor-Associated Dendritic Cells: Potential Impact on Antigen Cross-Presentation Machinery

**DOI:** 10.3390/cancers12092403

**Published:** 2020-08-24

**Authors:** Sven Burgdorf, Stefan Porubsky, Alexander Marx, Zoran V. Popovic

**Affiliations:** 1 Department of Cellular Immunology, LIMES Institute, University of Bonn, 53115 Bonn, Germany; burgdorf@uni-bonn.de; 2 Institute of Pathology, University Hospital, Johannes-Gutenberg University Mainz, 55131 Mainz, Germany; Stefan.Porubsky@unimedizin-mainz.de; 3 Institute of Pathology, University Hospital Mannheim, University of Heidelberg, 68167 Mannheim, Germany; Alexander.Marx@umm.de

**Keywords:** cancer acidity, hyperosmolarity, tumor microenvironment, cross-presentation

## Abstract

Macrophages (MΦ) and dendritic cells (DC), major players of the mononuclear phagocyte system (MoPh), are potent antigen presenting cells that steadily sense and respond to signals from the surrounding microenvironment, leading to either immunogenic or tolerogenic outcomes. Next to classical MHC-I/MHC-II antigen-presentation pathways described in the vast majority of cell types, a subset of MoPh (CD8^+^, XCR1^+^, CLEC9A^+^, BDCA3^+^ conventional DCs in human) is endowed with a high competence to cross-present external (engulfed) antigens on MHC-I molecules to CD8^+^ T-cells. This exceptional DC function is thought to be a crucial crossroad in cytotoxic antitumor immunity and has been extensively studied in the past decades. Biophysical and biochemical fingerprints of tumor micromilieus show significant spatiotemporal differences in comparison to non-neoplastic tissue. In tumors, low pH (mainly due to extracellular lactate accumulation via the Warburg effect and via glutaminolysis) and high oncotic and osmotic pressure (resulting from tumor debris, increased extracellular matrix components but in part also triggered by nutritive aspects) are—despite fluctuations and difficulties in measurement—likely the most constant general hallmarks of tumor microenvironment. Here, we focus on the influence of acidic and hypertonic micromilieu on the capacity of DCs to cross-present tumor-specific antigens. We discuss complex and in part controversial scientific data on the interference of these factors with to date reported mechanisms of antigen uptake, processing and cross-presentation, and we highlight their potential role in cancer immune escape and poor clinical response to DC vaccines.

## 1. The Role of Mononuclear Phagocytes in the Tumor Microenvironment

The mononuclear phagocyte system (MoPh) with its most important and most broadly explored players—macrophages (MΦ) and dendritic cells (DC)—comprises a major population of immune cells that migrate to and infiltrate tumor tissue. Crucial influence of continuous, highly plastic interaction between MoPh, tumor cells and surrounding milieu has been well-documented in the past decades [1]. The opposing role of different MΦ/DC subsets to this regard has highlighted the need to define (from a quantitative point of view) more and more ‘smaller’ genotypic, phenotypic and functional MoPh subgroups in order to decipher their possible role in antitumor immunity. The former strict differentiation between MΦ- and DC-expression profiles has meanwhile been updated and replaced by current concept based on the understanding that an expression signature and functional specialization of MoPh cells represents a rather unstable momentum that depends on interplay between cellular and molecular microenvironmental factors [2,3]. Due to an extraordinary instability and fluctuations in tumor microenvironment (TME), the categorization of tumor-associated MoPh profiles thus remains a challenging task. A number of scientific reports and excellent up-to-date reviews have addressed the question of receptor expression profiling of tumor-associated MΦ and DC in order to classify them functionally [4,5,6]. Thus, we do not intend to address MoPh classification algorithms; instead, we focus in this review on the cross-presentation by a subtype of tumor-associated DCs as an important functional link in triggering the cytotoxic antitumor response, and highlight the influence of low pH and high osmolarity of the TME on the cross-presenting capacities of these cells.

## 2. Modules of Efficient Antigen Presentation by MHC Molecules

Classical antigen-presentation pathways are not limited to professional phagocytes and imply two well-described antigen-presentation modules: (1) engulfment and processing of exogenous antigens for endosomal digestion and surface presentation in the complex with MHC-II-molecules to CD4^+^ T cells—an unspecific mechanism present in a vast majority of cell types; and (2) processing and presentation of endogenous (intracellular) antigens on MHC-I molecules in order to activate CD8^+^ T cell and induce their proliferation and cytotoxic response.

A third, special form of antigen-presentation can be seen (not exclusively, but most prominently) in a subset of DC: it is termed cross-presentation and is characterized as the capacity to present an external, phagocytosed antigen in the complex with MHC-I molecules to CD8^+^ T cells [7], thereby sensing the tumor- (or virus-) specific antigens and exposing them to induce an effective cytotoxic antitumor response [8].

### 2.1. Cross-Presenting Capacity of DC Subsets

Generally, DCs are categorized into conventional DCs (cDCs with previously defined cDC1 and cDC2 categories) and plasmacytoid DCs (pDCs), as reviewed elsewhere [9,10]. cDC2 (BDCA1^+^) have—especially in humans—at least a minor capacity to cross-present, even though there is evidence that cDC2 prefer classical MHC-II antigen presentation module [5,11]. By contrast, the role of cross-presentation by pDC is controversial: there are experiments showing intact clearance of viral antigens in a pDC-depleted system [12], indicating that pDCs likely do not have a major role in cross-presentation.

The cDC1 subtype seems to be the most potent cross-presenting DC population. The development of cDC1 has been reported to depend on IRF8- and Batf3- transcription factors and is characterized in humans by expression of the chemokine receptor XCR1, efficient uptake of apoptotic particles via CLEC9A (DNGR1) or necrotic debris via BDCA3 (CD141), prominent TLR3-reactivity with high IL-12 output, high intraendosomal reactive oxygen species and low acidification of endosomes [8,13,14]. In several animal models, the failure of cDC1-deficient mice to reject transplanted immunogenic tumors (for example, using Batf3-/- mice) underscores the importance of these cells in cross-presentation and consecutive CD8^+^ T cell priming [5,15]. Next, high expression of MHC-I pathway related genes has been reported as a hallmark of the cDC1 subset [16]. These cells also show enhanced expression of NADPH-oxidase 2 (NOX2), which has been linked to ROS production and active alkalization of endosomes, together with synchronous, low-level expression of the c-type lectin, Siglec-G (a potent NOX2-inhibitor). Both features are required for efficient cross-presentation and are critically responsible for the enhanced cross-presentation capacities of cDC1 cells [14,17].

Of relevance, compared to other intratumoral/peritumoral leukocytes, the cDC1 population is generally small in tumor tissue. Additionally, in contrast to the immunosuppressive role of tumor-associated macrophages (TAM), a high cDC1-load in malignant tumors has been shown to have a positive prognostic and predictive value [5,18]. Accordingly, if augmentation of the cytotoxic antitumor response through attraction of cDC1 to the cancer site and prolongation of their retention there is a therapeutic goal, induction of specific chemokine secretion by tumor cells and other immune cells (CCL4, CCL5, XCL1) might be the appropriate strategy [19,20]. Especially tumor-associated NK cells might be a key factor in this regard. Next to the aforementioned chemokines, NK cells secrete fms-like tyrosine kinase 3 ligand (FLT3-L) as well, which is regarded as a factor that prolongs cDC1 viability [5,21].

### 2.2. Subcellular Pathways of Cross-Presentation

On a subcellular level, two general cross-presentation pathways are described to date: the vacuolar pathway and the endosome-to-cytosol pathway [22,23]. In the vacuolar pathway, internalized antigens are degraded within endosomal compartments by lysosomal proteases and loaded onto MHC-I molecules there [24,25]. In the endosome-to-cytosol pathway, internalized antigens need to be translocated from endosomes into the cytosol, where they are degraded by the proteasome [26,27]. Afterwards, antigen-derived peptides are retranslocated by the transporter associated with antigen processing (TAP) into the ER or into endosomes for loading onto MHC-I [28,29,30]. Although both cross-presentation pathways have been well-documented in the past [31,32], most of the published data point to the endosome-to-cytosol pathway as a dominant mechanism of cross-presentation. The proteosomal antigen degradation seems to be a step of crucial importance for an efficient activation of CD8^+^ T cells to recognize and kill the target tumor cells or virus-carrying cells (reviewed in [8]). A recent study pointed out that proteasomes might also be present in antigen-containing endosomes and hence, proteasomal degradation might also play a role in the vacuolar cross-presentation pathway [33]. However, the physiological relevance of endosomal proteasomes still needs to be determined. In addition to the vacuolar and the endosome-to-cytosol pathway, ‘alternative’, endocytosis-independent mechanisms of cross-presentation have been suggested, such as the transfer of preprocessed antigens via a gap junction-meditated contact between a ‘donor cell’ and a DC [34] or ‘cross-dressing’, which assumes the acquisition of a peptide-loaded MHC-I molecule via membrane transfer [35]. Yet, the in vivo relevance of such models in the tumor setting remains unclear and needs further investigation.

## 3. Acidity and Hypertonicity as Biophysical Hallmarks of the TME

**Figure 1:**
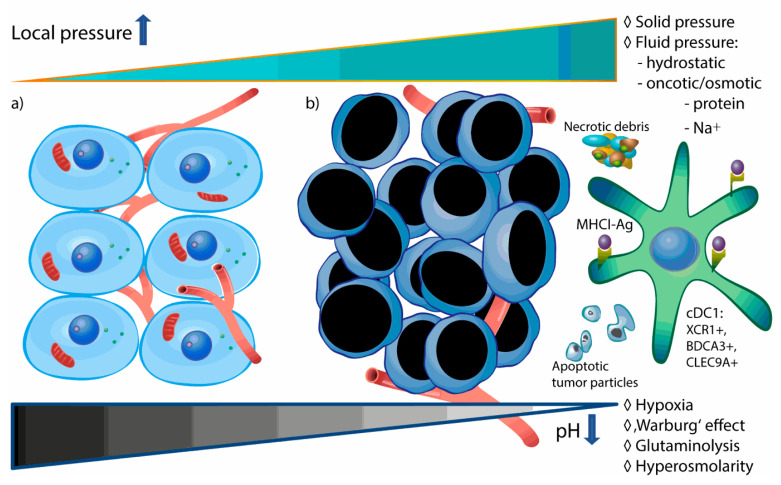
Typical microenvironment of tumor-associated cDC1 is acidic and hypertonic. In comparison to physiologic conditions (**a**), malignant neoplastic tissue (**b**) is characterized by lactate-induced decrease of pH value via hypoxia, aerobic glycolysis and glutaminolysis. On the other hand, increased (fluid and solid) pressure of tumor interstitium depends on biophysical characteristics of tumor mass, tumor cell death rate, abnormal blood supply but also nutritive aspects, like high-salt diet. Increased extracellular Na^+^ may further enhance lactate accumulation via supporting aerobic glycolysis.

In addition to varying biochemical and cellular parameters, physical stress plays an important role in tumor spreading and therapy response [36]. The typical microenvironment of solid tumors is characterized by hypoxia (mainly due to insufficient blood perfusion), low extracellular pH and high intratumoral pressure (Figure 1).

### 3.1. Mechanisms of TME Acidification

Acidity of the TME has been traditionally linked to hypoxia, as both phenomena are synchronous hallmarks of the TME. Briefly, normal human cells under aerobic conditions produce required amounts of ATP molecules from energy-rich glucose via cellular respiration process down to CO_2_ molecules resulting in the production of 32 molecules of ATP from one molecule of glucose. In normal cells under anaerobic conditions—but constitutively also in many tumors due to the Warburg effect (i.e., aerobic glycolysis; first described by Otto Warburg and his team in the 1923)—NADH is re-oxidized, leading to a reduction of pyruvate into lactate, the production of only two ATP molecules per molecule of glucose and resulting in local accumulation of lactate (reviewed in [37]). Thereby, even though the synthesis of lactate is not necessarily associated with hypoxic conditions, excessive lactate production and accumulation inside many tumors is responsible for their well described acidic TME. Specifically, melanomas, squamous cell carcinomas, breast cancer and many other adenocarcinomas as well as brain tumors show low pH values in their milieu, ranging from pH 5.8 to pH 7.4, as reviewed by Diaz et al. [38]. Other substrates next to glucose may also result in tumor-associated lactate production: for example, glutaminolysis pathway via citric acid cycle may be even a major source of lactate in cancer microenvironment [39]. Functionally, acidosis of the tumor interstitium has been shown to be a crucial factor of tumor survival, local progression and metastasis [40].

### 3.2. Elevated TME Pressure—Biophysical Prediction Models

On the other hand, very few reports are available on the role of high pressure in tumor micromilieus. It has been reported and reviewed that many solid tumors demonstrate an elevated interstitial pressure [1], forming a physical barrier to transcapillary transport and thereby affecting the antitumor therapy. Still, as a result of technical difficulties to directly measure intratumoral pressure in different tissue compartments, its relevance for tumor progression (involving immune escape mechanisms) has remained largely unexplored to date. In general, pressure-induced stress in tumors can be divided into two categories: fluid-phase and solid-phase stress. Further, fluid-phase stress can be roughly categorized into hydrostatic fluid pressure of the tumor interstitium and osmotic/oncotic pressure [41]. Increased osmotic pressure results from increased ionic and protein load of distinct etiologies. Limited available data in this regard demonstrate a general hypertonicity of some TMEs (for instance, in a pancreatic adenocarcinoma murine subcutaneous tumor graft model) [42,43,44]. In the absence of direct in vivo measurement techniques, a compelling biomechanical approach to predict an intratumoral osmotic pressure has been proposed [42]. In this approach, based on previously published data on increased hyaluron (and other glycosaminoglycan) content in melanomas, sarcomas and adenocarcinomas, the authors developed a triphasic mathematical and biomechanical model that takes into account solid and fluid tumor pressure together with transport of anions and cations. Interestingly, the authors suggest that increased hydraulic conductivity of the tumor-associated blood vessels (defined as interstitial fluid flow through the interstitial compartment [45]) elevates the intratumoral concentration of free ions and thereby osmotic pressure [42].

### 3.3. Potential Contribution of Increased Na^+^ Uptake to Hypertonicity of TME

Regulation of tissue osmolarity in regard to interstitial Na^+^ concentration has been extensively explored in the past decade. In order to understand and discuss a plausible Na^+^ accumulation in the tumor insterstitium, it is necessary to understand recent updates and challenges concerning the (patho)physiology of ‘central’ (renal) and peripheral regulation of Na^+^ metabolism:

First, historically, despite the documentation of interstitial storage of chloride in preclinical studies (observed already 1909 by Padtberg; reviewed in [46]) more than a century ago, for a long time it was assumed that the regulatory function of kidney in regard to Na^+^ keeps osmolarity of interstitium including peripheral tissue in a tight range, similar to that of plasma (290–300 mOsm/L). The concept of constant Na^+^ concentration of extracellular tissue has been challenged in the past years and pointed at alternative pathways of Na^+^ regulation, especially nonosmotic Na storage pools via electrolytic binding to sulfated glucosaminoglycans, i.e., important constituents of extracellular matrix. It has been proposed that this mechanism prevents water loss and buffers sodium concentration by balancing between free and stored (bound) Na^+^ [47]. In concordance with these observations, a recently developed MRI-based approach (Na-MRI) enabled precise detection of extracellular Na^+^ -fluctuations in time and space and revealed that peripheral tissue indeed does not maintain steady-state extracellular Na^+^ concentration [48]. As detected in subcutaneous tissue, patients with autoimmune disease, arterial hypertension, or renal failure show higher Na^+^ concentrations in skin in comparison to healthy counterparts [49,50].

Second, as mainly demonstrated in experimental animal models, a shift of nutritional habits towards high salt uptake may provoke a markedly increased Na^+^ content in peripheral tissues, independent on a putative preexisting pathologic condition [51,52].

## 4. Cross-Presentation Cascade in Acidic and Hypertonic Milieu: Current Data and Possible Implications

A successful cross-presentation of tumor antigen requires efficient binding and processing of engulfed material. The cascade of antigen processing (starting with antigen uptake, delayed degradation, translocation into the cytosol, transport and loading on MHC-I molecules and transport of endoplasmatic reticulum components to endosome) has been recently reviewed [8]. Therefore, we focus here on the scarce currently available data on pH- and Na^+^- dependent modifications of the cross-presentation pathway.

### 4.1. Cross-Presentation in Low Extracellular pH

#### 4.1.1. The Influence of Low pH on Antigen Uptake

DCs in the tumor micromilieu screen and bind soluble and particular antigens via a wide range of surface receptors. The abundancy and type of endocytic receptor in combination with the presented antigen likely have a significant influence on efficacy of cross-presentation per se. It is reasonable to assume, that binding and processing of apoptotic and necrotic tumor material via binding to different receptors like e.g., DEC205 will be a dominant mechanism of uptake in apoptotic particles-rich or necrotic tumors. DEC205, a member of mannose receptor family typically expressed on cross-presenting dendritic cells and on thymic epithelial cells [53,54], is a well-characterized endocytic receptor that may induce either tolerance or immunity, depending on external signals [55]. An in vitro study has shown a pH-dependent recognition of apoptotic particles and necrotic tumor cells by DEC205 via formation of a double-ringed receptor conformation in the acidic microenvironment, implying enhanced engulfment of tumor-associated material in acidic TME [53].

In dendritic cells, mannose receptor (MR) is another effective endocytic carbohydrate-binding damage-associated-molecular-pattern (DAMP) receptor with a variety of ligands (endogenous and microbial) [56]. MR has been reported to target the endocytosed material via pH-dependent steps for cross-presentation [57]. At physiological pH, the MR acquires an extended conformation. A decrease in pH (6–7, corresponding to early endosomes or pH 5–6 in late endosomes), results in a continuous conformational change of the receptor, which mediates ligand release [58]. Based on these data, it can be hypothesized that tumor-associated extracellular low pH decreases the capacity of MR to form stable receptor-antigen complexes and thus can be responsible for reduced cross-presentation in acidic micromilieu. Indeed, it has been shown that antigenic targeting of the human MR is capable of inducing antitumor immunity [59].

**Figure 2:**
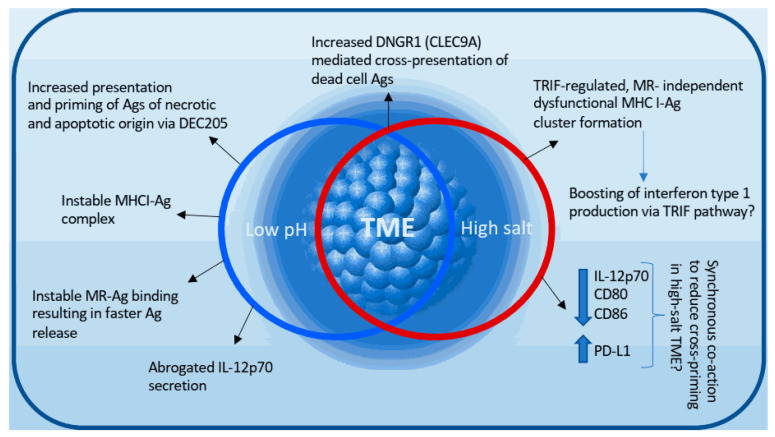
Impact of acidic and hyperosmolar tumor microenvironment (TME) on cross-presentation and cross-priming cascade is dynamic and complex. Interplay of low pH- and high Na^+^- triggered phenomena likely decides the fate and efficacy of cross-presentation. Also here can be postulated that tumor nature and viability (in regard to apoptotic and necrotic rate) partially shape the receptor–antigen binding signature and thus contribute to the response of cDC1 to biophysical TME stresses.

Of note, although both DEC205 and MR belong to the mannose receptor family, their pathways after antigen internalization seem to diverge. Whereas MR-mediated antigen engulfment in human DCs leads to its routing into early endosomes, delayed degradation and potent cross-presentation, internalization via the DEC205-pathway favors antigen-processing in lysosomes and rather poor cross-presentation [60]. Thus, summarizing these data, it can be discussed that an acidic cancer milieu may abrogate successful antigen internalization and processing within tumor-associated dendritic cell by favorizing less efficient DEC205-dependent cross-presentation model (Figure 2).

It is evident that low pH influences other DC receptors as well, not necessarily leading to a similar outcome, adding to the complexity of the phenomenon. An example is the pH- and ionic load- dependent alteration of another marker of type 1 classical DCs, DNGR1 [61]. DNGR1 (synonym: CLEC9A) is a DAMP-receptor typically expressed on cross-presenting DCs, facilitating cross-presentation of dead-cell-associated antigens. Hanc et al. have convincingly shown that low pH and increased ionic content of the microenvironment lead to a conformational change of the neck region and induction of so-called reduction-sensitive receptor dimers to trigger more efficient cross-presentation and cross-priming [61]. The detailed role of DNGR1 in regulation of the necrotic-cargo intracellular compartment [62] via directing the necrotic antigen material to nonlysosomal, rather alkaline, nondegradative niche, thereby facilitating CD8^+^ T cell activation [63] has been reviewed by Cueto et al. [64].

#### 4.1.2. The Influence of Low pH on Antigen-MHC-I Stability and Costimulatory Signals

Supporting the theory of damaged cross-presentation at low pH, older studies have documented that peptide-MHC-I complexes are more stable at neutral than at acidic pH [65]; still, no recent data are available to this regard. In addition, lactate has been reported to modulate cytokine secretion by monocyte-derived DCs. Lactic acid has triggered a significant reduction of IL-12p70 in tumor associated dendritic cells, hence blocking an important stimulatory signal in the cross-priming cascade [66,67]. In the same experimental setting no significant impact on secretion of ‘anti-inflammatory’ IL-10 could be seen.

Interestingly, both high salt and acidic milieu have been shown to trigger IL-1β secretion via activation of inflammasome pathway ([68]; also recent publication from Pitzer et al., FASEB, April 2020). Although recently published ovalbumine-based in vitro models suggest that NLRP3 inflammasome activation may trigger (MHC-I- and MHC-II- dependent) antigen presentation in general [69], its role in an efficient cross-presentation remains to be further investigated.

### 4.2. Cross-Presentation in Hyperosmolar Micromilieu

#### 4.2.1. Lessons from Murine Kidney and Cell Culture Models

The influence of hyperosmolarity via increased Na^+^ in the microenvironment of MoPh in inflammatory, non-neoplastic settings has been the subject of extensive studies in the past years [46,70,71,72,73,74,75,76,77]. Focusing on dendritic cells, our initial data from a murine kidney transplantation model have demonstrated a strong abrogation of cross-presentation pathway associated genes in the hypertonic renal medullary compartment [71]. Linking these results to function of DCs in hypertonic microenvironment that may reflect the osmolarity range of TME, our in vitro model of cross-presentation showed a significant reduction of cross-priming capacity in bone marrow derived dendritic cells (BMDCs) developed in hyperosmolarity [78]. Notably, the decreased cross-priming effect occurred despite increased uptake, processing and presentation of OVA-derived antigen in high salt conditions. In our experiments, blockade of cross-priming was a result of a TRIF-mediated (yet toll-like receptor-independent) dysfunctional MHC-I-peptide complex cluster formation. Having on mind the link between Interferon type 1 and TRIF signaling, as discussed by Jantsch et al. [46], it is possible that the Na^+^ induced, TRIF-mediated excessive expression of interferon type 1 reduces the cross-priming by DCs [79]. This hypothesis still requires further examination.

To our surprise, Na^+^-induced dysregulation of co-stimulatory and co-inhibitory molecules (including reduction of IL-12 secretion as well as upregulation of PD-L1 and inhibition of both CD80 and CD86 expression upon exposure to high salt) was not responsible for the reduction of cross-priming—at least not in a ‘single-parameter’-dependent manner. Nevertheless, we cannot exclude a coaction of the abovementioned regulatory molecules in high-salt induced blockade of cross-priming [78]. Intriguingly, the salt-induced cross-presentation phenotype was in this experimental setting independent of MR, suggesting that also the hypertonic TME may redirect MR-mediated antigen uptake towards other, in the light of cross-presentation less efficient DAMP receptors [78].

#### 4.2.2. Linking TME Hyperosmolarity to Lactate-Induced Acidosis

High salt content of TME may also modify cancer metabolism towards supporting aerobic glycolysis and consequent accumulation of lactate, highlighting the complexity of metabolic tumor surveillance [80]. It has been suggested that hypertonic extracellular stress induces the Warburg effect by enhancing glucose transport and lactic acidosis in tumor cells [81]; these results were concordant with older observations from breast cancer and liver tumor mouse models [82].

Taken together, limited and in part discordant published data indicate that interplay between acidic and hypertonic stress results in modification of cancer cell metabolism and dendritic cell function towards blockade of efficient cross-presentation and cross-priming (Figure 2).

## 5. Conclusions

Dendritic cells (specifically cDC1) are the most efficient antigen-presenting cells. Their unique cross-presentation capacity has been extensively explored in attempts to boost cytotoxic tumor immunity via DC vaccine strategies, albeit with rather poor clinical results to date. We reviewed here focused, limited and to some extent discordant data on the role of hypertonicity and acidity on DC function that largely go in line with other published observations regarding a general suppression of antitumor immunity via tumor metabolites [67,83,84].

In general, available evidence on the effects of low pH and hypertonicity on dendritic cells is (a) mainly focused on single steps of antigen uptake and presentation per se (to our knowledge without available data on subcellular antigen processing cascade) and thus poorly explored to date; (b) mostly related to in vitro or animal models, hence not automatically applicable to clinical situation and (c) in part based on older scientific studies that do not necessarily follow the current state of immunological knowledge. Specifically, methodological issues in measurement of pH and osmolarity of extracellular microenvironment in situ—in time and space—represent to our opinion the major obstacle to be addressed to this regard.

It must be pointed out that—independent of subcellular pathways—the effect of TME on immune cell activation should be finally observed in the context of tumor survival as the most relevant clinical endpoint. Clearly, antitumor immunity is a result of coaction of all parts of immune system at the tumor site and in regional draining lymph nodes and cannot be determined via observation of single components alone. Indeed, opposing data from two in vivo mouse tumor models based on subcutaneous injection of murine melanoma and lung carcinoma cells reported significant reduction of tumor growth upon high-salt intake via depletion of myeloid-derived suppressor cells [85]. On the other hand, in humans, highly malignant nature of tumors rising in hypertonic organ compartments (like collecting duct carcinoma of kidney and medullary renal cell cancer) with an extraordinary metastatic potential speak in favor of high salt-induced immune escape mechanisms. In the same line, a large set of epidemiologic data clearly indicates that high salt diet (independent on Helicobacter pylori infection) represents an independent high-risk factor for the development and progression of gastric cancer [86]. Nonetheless, mechanisms underlying the development and progression of potentially salt-induced malignancies remain unclear.

Finally, further interdisciplinary investigations of molecular and physical mechanisms of pH- and Na^+^-mediated modulation of DC function together with appreciation of dynamic, complex, species-, tissue- and tumor-type-specific relationships between immune system compartments and tumor microenvironments are necessary for a better understanding of and fighting against the immune escape of cancer.
